# Real-time Metagenomic Analysis of Undiagnosed Fever Cases Unveils a Yellow Fever Outbreak in Edo State, Nigeria

**DOI:** 10.1038/s41598-020-59880-w

**Published:** 2020-02-21

**Authors:** Fehintola V. Ajogbasile, Judith U. Oguzie, Paul E. Oluniyi, Philomena E. Eromon, Jessica N. Uwanibe, Samar B. Mehta, Katherine J. Siddle, Ikponmwosa Odia, Sarah M. Winnicki, Nosa Akpede, George Akpede, Sylvanus Okogbenin, Ephraim Ogbaini-Emovon, Bronwyn L. MacInnis, Onikepe A. Folarin, Kayvon Modjarrad, Stephen F. Schaffner, Oyewale Tomori, Chikwe Ihekweazu, Pardis C. Sabeti, Christian T. Happi

**Affiliations:** 1 African Centre of Excellence for Genomics of Infectious Diseases (ACEGID), Redeemer’s University, Ede, Osun State Nigeria; 2 Department of Biological Sciences, Faculty of Natural Sciences, Redeemer’s University, Ede, Osun State Nigeria; 3 Broad Institute of MIT and Harvard, Cambridge, Massachusetts USA; 4 Beth Israel Deaconess Medical Center, Division of Infectious Diseases, Boston, Massachusetts USA; 5 Center for Systems Biology, Department of Organismic and Evolutionary Biology, Harvard University, Cambridge, Massachusetts USA; 6 Institute of Lassa Fever Research and Control, Irrua Specialist Teaching Hospital, Irrua, Edo State Nigeria; 7 Department of Immunology and Infectious Diseases, Harvard T.H. Chan School of Public Health, Harvard University, Boston, Massachusetts USA; 8 Emerging Infectious Diseases Branch, Walter Reed Army Institute of Research, Silver Spring, MD USA; 9 Nigeria Center for Disease Control, Abuja, Nigeria; 10 Howard Hughes Medical Institute, Chevy Chase, Maryland USA

**Keywords:** Infectious-disease diagnostics, Pathogens, Phylogeny

## Abstract

Fifty patients with unexplained fever and poor outcomes presented at Irrua Specialist Teaching Hospital (ISTH) in Edo State, Nigeria, an area endemic for Lassa fever, between September 2018 - January 2019. After ruling out Lassa fever, plasma samples from these epidemiologically-linked cases were sent to the African Centre of Excellence for Genomics of Infectious Diseases (ACEGID), Redeemer’s University, Ede, Osun State, Nigeria, where we carried out metagenomic sequencing which implicated yellow fever virus (YFV) as the etiology of this outbreak. Twenty-nine of the 50 samples were confirmed positive for YFV by reverse transcriptase-quantitative polymerase chain reaction (RT-qPCR), 14 of which resulted in genome assembly. Maximum likelihood phylogenetic analysis revealed that these YFV sequences formed a tightly clustered clade more closely related to sequences from Senegal than sequences from earlier Nigerian isolates, suggesting that the YFV clade responsible for this outbreak in Edo State does not descend directly from the Nigerian YFV outbreaks of the last century, but instead reflects a broader diversity and dynamics of YFV in West Africa. Here we demonstrate the power of metagenomic sequencing for identifying ongoing outbreaks and their etiologies and informing real-time public health responses, resulting in accurate and prompt disease management and control.

## Introduction

Yellow fever (YF) is an acute mosquito-borne viral haemorrhagic fever that is endemic in forty-seven countries across Africa, Central and South America. A 2013 mathematical model study using African data from the previous 25 years estimated that the burden of severe YF ranged from 84 000–170 000 cases with 29 000–60 000 deaths^1^. Once common in Nigeria^2^ YF was largely absent for twenty years, until 2017 when a new and still ongoing outbreak was reported^3^. A total of 112 confirmed cases with 11 deaths have been recorded between September 2017 and March 2019, prompting the vaccination of more than 10 million people in the affected states^4^. However, the relative absence of yellow fever (YF) in Nigeria over prior years led to unintended consequences of reducing vigilance for a disease whose clinical presentation overlaps other endemic infections such as malaria and Lassa fever. The signs and symptoms of YF include fever, myalgia, arthralgia, jaundice and mucosal hemorrhage^5^, with case fatality among reported cases estimated between 15–50%. Definitive diagnosis during early stages is made by detection of viral RNA via quantitative polymerase chain reaction (qPCR) in blood and urine samples, while in later stages viremia typically stops and diagnosis is serological^6^. There is no specific treatment, although YF is preventable using a single dose of yellow fever vaccine, with high initial seroconversion rate (usually >90%) and >75% persistent titers for several years after primary vaccination^7^.

In the last quarter of 2018, clinicians at Irrua Specialist Teaching Hospital in Edo State, Nigeria, a region endemic for Lassa fever, identified a cluster of patients with severe febrile illness who tested negative for Lassa virus. Here we report the use of metagenomic sequencing to identify the causal agent of this outbreak as YFV, explore the genetic diversity and infer the recent history of this viral pathogen in Nigeria.

## Results

### Demographics

Of the 50 samples tested, 31(62%) were male and 14(28%) were female. Their ages ranged from 3 to 60 years with a mean age of 19.4 years (Table 1).

**Table 1:** Demographics for n = 50 tested samples; percentages in parentheses.

Gender	male	female					no data
	31 (62)	14 (28)					5 (10)
Age range	0–10	11–20	21–30	31–40	41–50	51–60	no data
	13 (26)	15 (30)	10 (20)	3 (6)	0 (0)	3 (6)	5 (10)

### SYBR based YFV qRT-PCR

Twenty-nine out of the fifty plasma samples were found positive for YFV by RT-qPCR (Supplementary Table S1).

### Phylogenetic analysis

**Figure 1:**
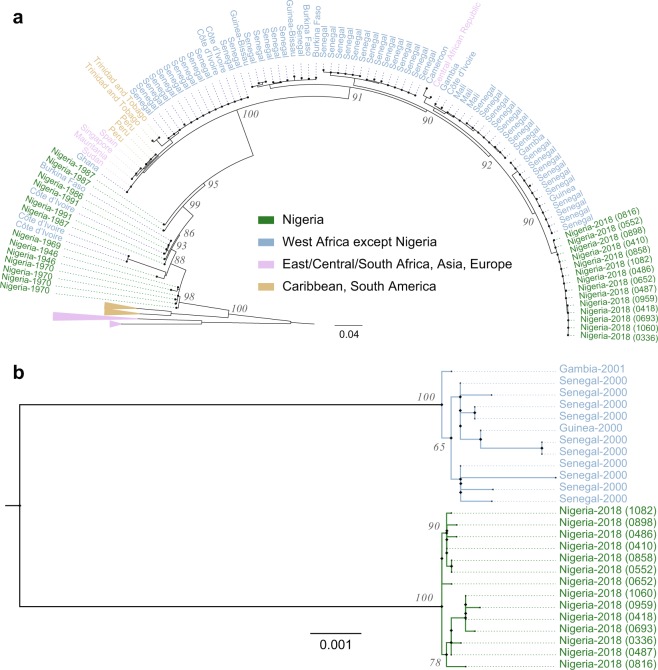
Maximum likelihood phylogenetic trees for YFV, with bootstrap values shown for major branches. (**a**) 216 prM/E gene sequences (**b**) 2018 Nigerian YFV whole genome sequences and the West African whole genome sequences with which they most closely cluster. ID numbers for 2018 Nigerian sequences correspond to those given in Fig. 2; GenBank accession numbers are listed in Supplemental Table 1. Scale bars are in units substitutions per site. Trees were inferred using IQTREE and FigTree was used to view and annotate the trees. Cosmetic adjustments were made in Adobe Illustrator CC.

We assembled fourteen YFV genomes mostly from samples with lower Ct values as these had sufficient YFV reads for genome assembly. Maximum likelihood phylogenetic analysis revealed that the 2018 YFV sequences formed a tightly clustered clade (Fig. 1a), more closely related to sequences from other West African countries (mean pairwise identity = 95.2%) than to earlier (1946–1991) Nigerian sequences (mean pairwise identity = 91.4%). Bayesian analysis estimated the tMRCA of the fourteen (14) 2018 sequences from Nigeria as 3 years (95% HPD interval), while the tMRCA of the pre-1991 sequences from Nigeria is 195 years (95% HPD interval) (Supplementary Fig. S1).

### Metagenomic analysis

**Figure 2:**
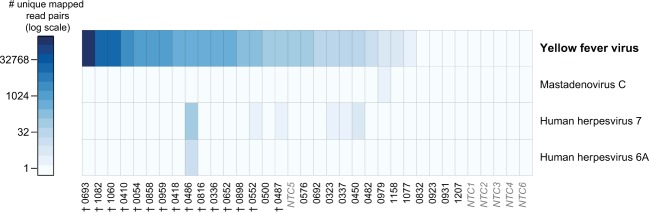
Virus detection by metagenomic sequencing. Heatmap shows the number of deduplicated read pairs that mapped to the indicated viral RefSeq genome; all viral species where at least one sample produced reads that aligned to a viral RefSeq genome are shown. Detected species were first filtered using Kraken. † samples from which we were subsequently able to assemble YFV genomes.

Metagenomic analysis revealed YFV as the only virus with reads from across the viral genome and appearing in multiple samples. Other viruses with small numbers of reads suggested by Kraken classification were human herpesvirus 6A, human herpesvirus 7 and mastadenovirus C (Fig. 2). The reads initially classified as herpesvirus, however, consisted of oligonucleotide repeats with weak similarity to terminal regions of the betaherpesvirus genome. These reads are, however, non-specific, and match several other genera in a standard NCBI BLAST search (after removing the low-complexity filter), and they therefore likely represent false Kraken positive classification of nonspecific template with tandem repeats. In contrast, mastadenovirus C reads did appear to be specific matches, but they totaled only three read-pairs in a single sample.

We separately noted that one of the 6 negative (water) controls — labeled NTC5 in Fig. 2 — contains a small number of reads from YFV. Only one sample (0054) processed in the same batch as NTC5 was positive for YFV, and this sample was positive by qPCR before being processed for sequencing. This argues that 0054 was a true positive that likely contaminated the negative control. However, to remove any ambiguity, we did not further include 0054 in the case counts or phylogenetic analyses reported here.

## Discussion

We established the presence of YFV in Edo State within three days of receiving initial samples, and we shared this information immediately with the referring hospital (ISTH) and the Nigeria Center for Disease Control (NCDC). Based in part on these findings, NCDC and the Nigeria Federal Ministry of Health declared an outbreak in Edo state the following day^4^, prompting more samples to be sent for diagnosis.

Notably, these are the first sequence data reported to date of recent Nigerian YFV cases and the only complete Nigerian YFV genomes from patient samples collected after 1950. Our phylogenetic analysis suggests that the YFV clade responsible for the 2018 outbreak in Edo State does not descend directly from the Nigerian YFV outbreaks of the last century but is instead part of the broader diversity of YFV in West Africa. These results are supported by the few available historical whole genome sequences (Fig. 1b), but a detailed understanding of the recent yellow fever outbreak in Nigeria will require sequencing of stored samples from other states and from the past decades.

The parallel detection of yellow fever on unbiased metagenomic sequencing of multiple samples illustrates the power of genomics in explaining a suspected outbreak. In circumstances where sample collection is unplanned, simultaneous analysis of multiple samples can improve the sensitivity for the true etiology; in this case, we suspect the short period of viraemia relative to the duration of symptoms in YF^8^ partly explains the absence of detectable viral nucleic acid in a subset of samples.

This ability to rapidly identify and characterize a re-emerging virus – in an unusual cluster identified by local health officials – highlights the value of in-country genomics capacity. Serology using ELISA is the current method of choice for yellow fever diagnosis by the WHO. This diagnosis method despite its limitations is done in very few selected laboratories and cost about one thousand US dollars per sample. The integration of genomics capacity into the established, but siloed, pathogen-specific diagnostic platforms developed over the past 20 years provides exciting opportunities for public health surveillance.

## Materials and Methods

### Sample collection and testing

In 2018 clinicians and public health authorities at Irrua Specialist Teaching Hospital (ISTH) in Edo State, Nigeria noted a pattern among a series of fifty (50) patients who had common clinical presentations, poor outcomes, a lack of clear diagnosis and resident in contiguous local government areas. All patient samples tested negative for Lassa virus by RT-qPCR. Aliquots of plasma were sent to the African Center of Excellence for Genomics of Infectious Disease (ACEGID) at Redeemer’s University for further genomic investigation.

### Sample preparation and sequencing

Plasma samples were inactivated in AVL and viral RNA was extracted according to the QiAmp viral RNA mini kit (Qiagen) manufacturer’s instructions. Extracted RNA was treated with Turbo DNase to remove contaminating DNA, followed by cDNA synthesis with random hexamers. Sequencing libraries were prepared using the Nextera XT kit (Illumina) as previously described^9^ and sequenced on the Illumina Miseq platform with 101 base pair paired-end reads.

### SYBR Based YFV qRT-PCR

SYBR green RT-qPCR was performed on a Roche LightCycler 96; samples were called positive if at least two of three triplicate reactions showed any evidence of amplification. Briefly, 3 μL of RNA was used per reaction as a template for amplification, and this sample was added to 7 μL of reaction mixture containing 1.32 μL of H_2_O, 5 μL of Power Sybr master mix, 0.08 μL of 125X reaction mix and 0.3 μL sense and anti-sense primers. Real-time RT-qPCR amplification was carried out for 45 cycles at 48 °C for 30 min, 95 °C for 10 min, 95 °C for 15 sec, and 60 °C for 30 sec. Temperatures for the melt curves were 95 °C for 15 sec, 55 °C for 15 sec and 95 °C for 15 sec. The YFV primer sequences have been published elsewhere^8^.

### Metagenomic analysis of viral Infections

Kraken^10^ was used to perform an initial taxonomic classification of all viral taxa present in the sample using a database that encompassed the known diversity of all viruses that infect humans, as previously described^11^. Alignment to reference genomes was performed for each virus species identified by Kraken as present in one or more samples in order to confirm and obtain de-duplicated counts of classified reads. Alignment was performed with Novoalign (http://www.novocraft.com) using the following parameters: “-r Random -l 30 -g 40 -x 20 -t 502” and Picard (http://broadinstitute.github.io/picard) was used to mark and remove duplicates.

### Genome assembly and maximum likelihood phylogenetic analysis

Viral genomes were assembled using viral-ngs v1.21.2^12,13^ on the DNAnexus platform, and MAFFT v7.310^14^ was used to align these genomes with all African YFV genomes available in GenBank as of 19th January 2019 (including a small number of non-African sequences as an outgroup). Using Geneious 2019.0.4^15^, 678 bp of the prM/E region of the genome was identified as having the most coverage by earlier GenBank sequences from Africa; this region was extracted and used to infer a maximum likelihood tree using IQTREE v1.5.5^16^. We used a Tamura-Nei nucleotide-substitution model with a gamma distribution of rate variation among sites^17^ and ultrafast bootstrapping^18^. For each pair of aligned 678 bp prM/E regions, we calculated a percent nucleotide identity. We then averaged this value over all pairs in a given branch of the maximum likelihood tree to report “mean pairwise identity” within a putative clade.

### Bayesian phylogenetic analysis

Time-scaled Bayesian phylogenetic analysis was carried out using a Markov chain Monte Carlo (MCMC) algorithm implemented in the BEAST v1.10.4^19^ package with BEAGLE^20^ to improve run-time. The evolutionary and demographic processes were estimated from the sampling dates of the prM/E sequences using a model that incorporated a General Time Reversible (GTR) + Gamma distribution (four categories) model with “(1 + 2),3” codon partitioning, an uncorrelated relaxed clock with log-normal distribution^21^, and a Bayesian skyline coalescent tree prior distribution^22^. All Bayesian analyses were run for 200 million MCMC steps, with parameters and trees sampled every 10000 generations. The uncertainty in our parameter estimates was assessed by calculating the effective sample size (ESS) and the 95% highest probability density (HPD) values using the TRACER v1.6.0^23^ program. Maximum clade credibility (MCC) trees summarizing all MCMC samples were generated by TreeAnnotator v1.10.4^24^ software, with a burn-in rate of 10%. FigTree v1.4.4^25^ was used to view and annotate the MCC tree. This analysis used the same 678 bp of the prM/E region of the YFV genome as was used for the maximum likelihood approach above, possibly biasing our time to most common recent ancestor (tMRCA) estimates.

### Ethical approval

The study was conducted in accordance with the Declaration of Helsinki, and the protocol was approved by the Institutional Review Boards of ISTH (Edo State, Nigeria), Redeemer’s University (Osun State, Nigeria) and Harvard University (Massachusetts, USA). Excess plasma samples used for clinical testing were obtained under a waiver of consent granted by the ISTH Research Ethics Committee (ISTHREC).

## Data Availability

All sequences from this study are available on GenBank with accession numbers MK457700 - MK457703 and MN211301 - MN211311.

## Supporting Information

Supporting Information.
