# Supplementary material for: Real-time Metagenomic Analysis of Undiagnosed Fever Cases Unveils a Yellow Fever Outbreak in Edo State, Nigeria
Source: Sci Rep. 2020 Feb 21;10:3180. doi: 10.1038/s41598-020-59880-w (PMC7035389; doi:10.1038/s41598-020-59880-w)
Supplement: Supplementary file 1 — Supporting Information. [file 41598_2020_59880_MOESM1_ESM.pdf]

Real-time Metagenomic Analysis of Undiagnosed Fever Cases Unveils a Yellow Fever Outbreak in Edo State, Nigeria

Fehintola V. Ajogbasile [1,2,*], Judith U. Oguzie [1,2*], Paul E. Oluniyi [1,2*], Philomena E. Eromon [1*], Jessica N. Uwanibe [1,2*], Samar B. Mehta [3,4], Katherine J. Siddle [3,5], Ikponmwosa Odia [6], Sarah M. Winnicki [3], Nosa Akpede [6], George Akpede [6], Sylvanus Okogbenin [6], Ephraim Ogbaini-Emovon [6], Bronwyn L. MacInnis [3,7], Onikepe A. Folarin [1,2], Kayvon Modjarrad[8], Stephen F. Schaffner [3,5,7], Oyewale Tomori [1], Chikwe Ihekweazu [9], Pardis C. Sabeti [3,5,7,10], Christian T. Happi [1,2,6,7,**]

1. African Centre of Excellence for Genomics of Infectious Diseases (ACEGID), Redeemer's University, Ede, Osun State, Nigeria. 2. Department of Biological Sciences, Faculty of Natural Sciences, Redeemer's University, Ede, Osun State, Nigeria. 3. Broad Institute of MIT and Harvard, Cambridge, Massachusetts, USA. 4. Beth Israel Deaconess Medical Center, Division of Infectious Diseases, Boston, Massachusetts, USA. 5. Center for Systems Biology, Department of Organismic and Evolutionary Biology, Harvard University, Cambridge, Massachusetts, USA. 6. Institute of Lassa Fever Research and Control, Irrua Specialist Teaching Hospital, Irrua, Edo State, Nigeria. 7. Department of Immunology and Infectious Diseases, Harvard T.H. Chan School of Public Health, Harvard University, Boston, Massachusetts, USA. 8. Emerging Infectious Diseases Branch, Walter Reed Army Institute of Research, Silver Spring, MD, USA. 9. Nigeria Center for Disease Control, Abuja, Nigeria. 10. Howard Hughes Medical Institute, Chevy Chase, Maryland, USA.

*These authors contributed equally, **Corresponding author: happic@run.edu.ng

SUPPLEMENTARY SECTION

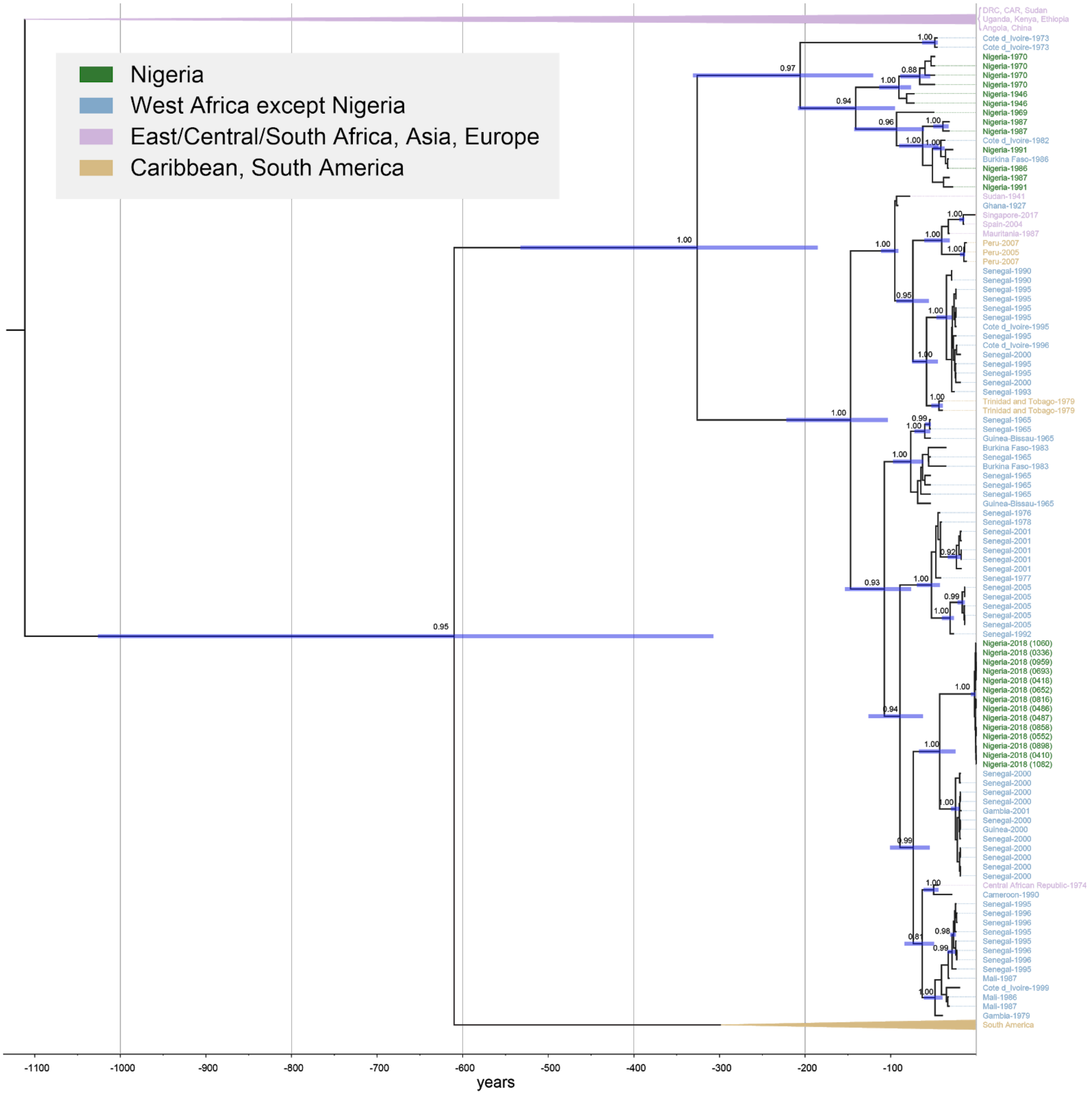

Supplementary Figure S1: Time-scaled Bayesian MCC phylogenetic tree for a region of the prM/E gene of 216 YFV sequences, aligned using MAFFT. The tMRCA of the fifteen (14) 2018 sequences from Nigeria is 3 years, 95% HPD interval [1, 7], while the tMRCA of the pre-1991 sequences from Nigeria is 215years, 95% HPD interval [121, 331]. All posterior probabilities over 0.80 are shown to the upper left of the associated branch point, along with a purple bar marking the associated 95% HPD interval.

Supplementary Table S1: **Yellow fever RT-qPCR & sequencing results**

Sample ID	GenBank accession #	YFV RT-qPCR C_t (cycles)	Estimated YFV RT-qPCR concentration (copies/µl)	YFV deduplicated read pairs aligned	Total read pairs sequenced
0693‡	MN211306	21.85	533000	555703	1974510
1060‡	MN211311	30.73	8150	36206	808444
1082‡	MN211301	31.03	7118	37011	1850149
0959‡	MN211302	34.12	1626	1873	596360
0858‡	MN211304	31.97	3968	2154	1771347
0418‡	MN211309	30.29	1609	3067	813891
0054‡*	pending	26.15	16526	**2379***	1212206
0410‡	MN211310	26.32	14944	3041	2353001
0652‡	MN211307	32.85	2584	479	3463575
0336‡	MK457700	34.72	247	568	2028788
0487†	MK457702	35.62	147	138	653380
0692		36.18	523	79	1822472
0486‡	MN211308	25.06	31800	808	5113134
0816‡	MN211305	30.40	8476	805	740066
0552†	MK457703	35.02	212	203	802365
0898‡	MN211303	29.43	13552	455	2913828
0450		33.94	388	46	651410
0500		30.46	1267	139	1147117
0323		34.05	154	62	1339359
0576		30.11	1542	87	1862063
0337		25.57	23380	48	2759890
0482		31.72	594	21	1229711
0832		36.54	437	1	1882812

0979		35.23	956	6	1733372
1158		35.28	935	5	2241764
0923		33.67	2016	1	2522614
1077		36.90	436	2	1813794
0931		31.62	5372	0	2692537
1207		36.38	557	0	2345025
NTC1		-	-	0	365834
NTC2		-	-	0	457056
NTC3		-	-	0	557561
NTC4		-	-	0	25994
NTC5		-	-	88	38939
NTC6		-	-	0	460467

‡: complete genome assembled; †: partial genome assembled.

* Sample ID 0054 was initially positive by qPCR with a C_t of 26. The same sample subsequently showed 2379 unique reads that mapped to YFV. As discussed in the main text, we excluded it from further phylogenetic analysis due to possible contamination with the negative control in its batch (NTC5 above, 88 unique reads mapping to YFV).
